# SVIoT: A Secure Visual-IoT Framework for Smart Healthcare

**DOI:** 10.3390/s22051773

**Published:** 2022-02-24

**Authors:** Javaid A. Kaw, Solihah Gull, Shabir A. Parah

**Affiliations:** Department of Electronics and Instrumentation Technology, University of Kashmir, Srinagar 190006, India; javaidkaw@gmail.com (J.A.K.); solihah.scholar@kashmiruniversity.net (S.G.)

**Keywords:** visual-IoT, reversibility, information hiding, bandwidth, security, computational complexity

## Abstract

The advancement of the Internet of Things (IoT) has transfigured the overlay of the physical world by superimposing digital information in various sectors, including smart cities, industry, healthcare, etc. Among the various shared information, visual data are an insensible part of smart cities, especially in healthcare. As a result, visual-IoT research is gathering momentum. In visual-IoT, visual sensors, such as cameras, collect critical multimedia information about industries, healthcare, shopping, autonomous vehicles, crowd management, etc. In healthcare, patient-related data are captured and then transmitted via insecure transmission lines. The security of this data are of paramount importance. Besides the fact that visual data requires a large bandwidth, the gap between communication and computation is an additional challenge for visual IoT system development. In this paper, we present SVIoT, a Secure Visual-IoT framework, which addresses the issues of both data security and resource constraints in IoT-based healthcare. This was achieved by proposing a novel reversible data hiding (RDH) scheme based on One Dimensional Neighborhood Mean Interpolation (ODNMI). The use of ODNMI reduces the computational complexity and storage/bandwidth requirements by 50 percent. We upscaled the original image from M × N to M ± 2N, dissimilar to conventional interpolation methods, wherein images are upscaled to 2M × 2N. We made use of an innovative mechanism, Left Data Shifting (LDS), before embedding data in the cover image. Before embedding the data, we encrypted it using an AES-128 encryption algorithm to offer additional security. The use of LDS ensures better perceptual quality at a relatively high payload. We achieved an average PSNR of 43 dB for a payload of 1.5 bpp (bits per pixel). In addition, we embedded a fragile watermark in the cover image to ensure authentication of the received content.

## 1. Introduction

The technological advances in communication and networking infrastructure in the past few decades have resulted in an exponential increase in digitization. The emergence of new technological concepts, such as the Visual Internet of Things (VIoT) and cloud computing has forced the potential applicability of technology in areas such as smart-healthcare, smart home, smart industry, etc. [1,2]. The present trend, in areas such as smart-health, is to collect the digitized information from numerous devices and/or IoT nodes and exchange said information through various networking platforms to achieve high-performance efficiency in terms of speed, resources, and cost factor [3]. Unfortunately, utilizing insecure networks, such as the internet, etc., for transferring critical data has been found to face continuous threats from the adversary. The threat of cyberattacks on privacy and data integrity faced by service providers has been on the rise. Take the example of the medical field; the data breaches associated with the healthcare sector have grown exponentially in recent years, and millions of health records are breached every year [4]. According to the Protenus Breach Barometer, more than 40 million patient data records were breached in 2021, of which 8 million records were breached by insiders, and 758 breaches were incidental [5]. In addition, it was reported that there was a 42% hike in these data breaches compared to the previous year [5].

The situation, therefore, demands the development of comprehensive security, privacy, and authentication solutions for IoT-driven systems, owing to their dynamic and resource-constrained nature.

**Figure 1:**
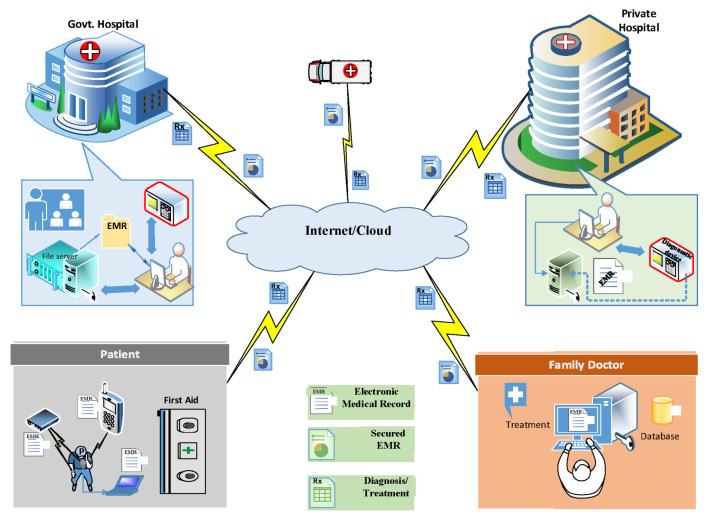
A typical smart-health system.

One of the common solutions adopted is the revisitation of various well know encryption algorithms to develop their lightweight counterparts for IoT-based platforms. However, given the nature and magnitude of security issues, the development of sustainable security solutions for critical applications, such as health care and smart cities, is of the utmost importance [6,7]. One of the possible solutions that has attracted attention is the embedding of information in images. This technology not only provides security, but is also capable of exactly identifying a node in a distributed computing scenario, such as IoT-driven networks, e.g., smart-health in a smart city. An IoT-driven smart-health setup is presented in Figure 1.

As described in Figure 1, multiple devices are needed to exchange the Electronic Medical Record (EMR) for proper diagnosis/treatment remotely. The EMR, related to a particular medical condition, is collected through various devices and sensors monitoring the patient. Instead of transmitting the EMR through a highly insecure network, the information hiding technology embeds the EMR in the patient’s medical imagery, to put the adversary at bay. The transmitted imagery-containing data are received by different authentic receivers for diagnosis and prescription.

Information hiding has been widely studied in both academia and industry in recent years. The main research directions for data hiding include copyright identification, data integrity, authentication, tamper recovery, retrieval of original images, and covert communication [8,9,10]. Information hiding could be reversible, as well as irreversible. Reversible hiding is suitable for applications such as smart-health, etc., where recovery of the cover image at the receiver is important [11,12,13]. One of the main challenges in RDH is the payload. Many high-payload RDH solutions, based on interpolation, exist; however, the techniques based on Image interpolation add significant redundancy, making them unfit for resource-constrained platforms such as IoT, as they require more storage space/bandwidth. In this paper, we present SVIoT, which makes use of novel One Dimensional Neighbor Mean Interpolation (ODNMI). The use of ODNMI in SVIoT reduces the computational complexity and storage/bandwidth requirement by 50%. This makes SVIoT a superior candidate for securing visual data in IoT-based multimedia systems. Moreover, the proposed scheme is capable of detecting tamper at the receiver, and as such is capable of authenticating the content. The main contributions of this paper are:

1. We propose an efficient security framework, SVIoT, based on the novel concept of ODNMI.
2. The use of ODNMI in SVIoT ensures reduction of computational complexity and storage/bandwidth requirements by 50 percent.
3. We make use of an innovative mechanism of Left Data Shifting (LDS) before embedding data, to ensure better stego-image quality for high embedding rates.

The rest of the paper is structured as follows. Section 2 describes the related work. The proposed work is described in detail in Section 3. Results and discussions are presented in Section 4. The paper concludes in Section 5.

## 2. Related Literature

IoT-based services have become an indispensable part of smart-city services. They have applications in healthcare, maintenance, autonomous vehicles, etc. Visual-IoT infrastructure requires large bandwidth, resulting in a challenging gap between computation and communication. Moreover, the data exchanged is highly vulnerable to attacks. To ensure better security, and lesser bandwidth requirements, data hiding could be used. Some of the data hiding works that could be used, in visual-IoT in general and smart-health in particular, are presented as follows.

Conventional data-hiding techniques, such as Least Significant Bit (LSB) substitution [14], LSB matching [15], and Pixel Value Differences (PVD), result in cover media distortion, which remains even after the hidden data are extracted. Though these irreversible data-hiding techniques provide improved data-embedding capabilities, they cannot be used in applications were extraction of both the secret data and the original image is the prime requirement. Examples of such cases include military applications, digital archiving, medical diagnosis, and law enforcement, wherein it is critical to reverse the marked media back to the original cover media after retrieving the hidden data.

Reversible Data Hiding (RDH) is used to ensure perfect retrieval of the cover image from stego-image, and as such is used for critical applications, such as healthcare. RDH is being studied with vigor by the research community, to attain the futuristic and sustainable goals of secure and reliable smart-healthcare setups. Difference Expansion (DE) [16], Prediction Error Expansion [17,18], and Histogram bin shifting (HBS) [19,20,21] have been used for developing RDH schemes. However, the schemes based on DE and HBS are characterized by low embedding capacity. Nature-inspired computing-based RDH schemes, based on the Genetic Algorithm (GA) and Particle Swarm Optimization (PSO), have been reported in [22,23]. These schemes are computationally complex, and handle less payload. Low embedding capacity techniques are not feasible in bandlimited environments. This is because of the fact that the secret data would require a large number of cover images for secure communication, increasing bandlimited channel load and reducing throughput.

Maintaining reversibility and ensuring high payload is of paramount importance in a visual-IoT-driven smart-healthcare system. Interpolation-based RDH schemes have been found to have a high embedding capacity. The majority of interpolation-based RDH schemes firstly downscale a 2N × 2M image to an N × M image, which is interpolated to the size of 2N × 2M. Some prominent examples in this direction include Interpolation by Neighboring Pixels (INP) [24], Neighborhood Mean Interpolation (NMI) [25], the Pixel Repetition Method (PRM), the Pixel Permutation Method (PPM) [26], and Optimal Pixel Repetition (OPR) [27]. The first interpolation-based RDH scheme using NMI was proposed by Jung and Yoo [25]. This scheme is capable of hiding data reversibly, as well as irreversibly, and provides a PSNR of above 35 dB for a payload of 0.96 bpp. It was later modified by Abadi et al. [28] using histogram modification, with an increase in payload. Marginal improvements to NMI [25] were seen in [29]. Lee and Huang [24] proposed another method, called Interpolation using Neighboring Pixels (INP), with a higher data-embedding capacity. In INP and NMI, simple operations are executed on the seed pixels, such as calculating the mean of the two neighboring pixels so that newly created pixels bear a resemblance to their neighboring pixels. PRM repeats the seed pixel, and hence results in a 2 × 2 block from one seed pixel, while in PPM, some basic mathematical operations are used to create new pixels, which are permuted pixels for data embedding as per [26]. In OPR [27], the seed pixels are used to obtain the optimal values of embedding pixels. This scheme has been tested for a wide variety of images, such as medical images, texture images, etc. In [30], a reversible data-hiding scheme based on Pixel to Block (PTB) conversion was presented. The proposed scheme is an effective and computationally efficient alternative to various existing interpolation-based schemes. It provides better quality watermarked images, with an average PSNR of 43.36 dB, for a payload of 0.75 bpp.

From the literature, it is evident that various schemes have been developed in the context of RDH using interpolation techniques, including INP, NMI, PRM, and PPM. These methods increase the image size by a factor of four, thus resulting in a higher data throughput, which in turn demands larger storage space and/or larger bandwidth and leads to increased system complexity; as such, these schemes are not ideal for use in visual-IoT systems as methods of providing security.

Towards this end, we propose an ODNMI scheme, with a special focus on reducing data redundancy leading to reduced storage space and bandwidth requirements, while maintaining reversibility, along with high embedding capacity. Data collected via continuously sensing IoT-based smart sensors needs to be transmitted via insecure communication channels. This information pertains to patients, and is sensitive, demanding high end security. In the proposed method, this data are embedded into the interpolated image using LDS. Further, to enhance the security of the secret data, it is encrypted using an AES-128 encryption algorithm. This data, along with the original image, can be recovered without loss at the receiver. The ODNMI scheme provides the advantage of a reduction in bandwidth/storage space by half, by generating a cover image of dimensions M × 2N from an input image of size M × N, instead of 2M × 2N.

## 3. Proposed Framework: SVIoT

**Figure 2:**
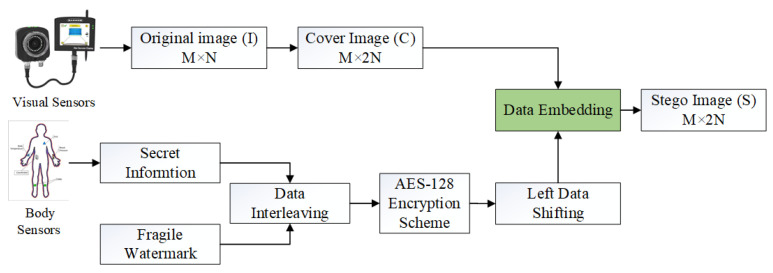
Block diagram of the proposed scheme.

In a smart healthcare-based setup, IoT nodes are deployed for the transmission of acquired data to a remote doctor. The transmission of this data over insecure communication networks can bring forth security, privacy, and content integrity issues. In such a scenario, the development of novel security mechanisms for IoT-driven networks is necessary. Towards this end, we developed a novel security mechanism, called SVIoT, for the transmission of patient information in an IoT-based healthcare setup. It requires relatively reduced resources, such as memory and bandwidth, which is in tune with IoT-setup requirements. The main purpose of the SVIoT is to ensure reversibility, privacy, and data integrity, by hiding the sensitive data in a cover image before it is transmitted through an insecure network in the IoT setup. The block diagram of the proposed SVIoT scheme is shown in Figure 2. Firstly, Visual-IoT sensors are used to collect the information (image data). This image is then used to carry patient information, comprised of details including blood pressure, heart rate, ECG, etc., obtained by a set of IoT sensors. The patient’s personal information, such as patient name, age, sex, ID, insurance, etc., is appended to it. This information capsule of details comprises our secret data. The secret data are first encrypted, using an AES-128 encryption algorithm, and is then embedded in the patient’s medical images. This is followed by embedding it in the cover image generated by ODNMI. Section 3.1 describes ODNMI in detail. To ensure better imperceptibility of watermarked images, we make use of LDS on the data to be embedded. Section 3.2 provides a brief description of LDS. Data embedding is discussed in Section 3.3, while data extraction is presented in Section 3.4.

### 3.1. One Dimensional Neighbor Mean Interpolation (ODNMI)

Jung and Yoo [23] proposed embedding a secret message in a cover image generated by the NMI method, wherein a two-dimensional image, of size M × N, is upscaled to 2M × 2N, hence increasing the size of the generated cover image to four times that of the original image. This extra burden on the network/storage devices is reduced by the proposed ODNMI technique. Let “*I*” represent the original/seed image, and “*C*” represents the cover image. Corresponding to every pixel of the seed image, we generate one new pixel using ODNMI. The generation of each new pixel in the cover image C (*I*, *j* + 1), from the corresponding seed pixel *I* (*i*, *j*) in the original image, using ODNMI, is governed by the following set of equations:

(1) C(i, 2j−1)=I(i,j) 

(2) $$C\left(i,2j\right)=Ceil\left[\frac{\left[I\left(i,j\right)+I\left(i,j+1\right)\right]}{2}\right]For\;j<N$$

(3) C(i,2j)=I(i,j) For j=N

**Figure 3:**
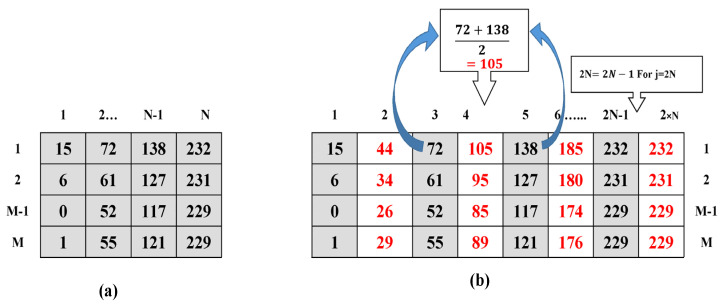
(**a**) Original image block; (**b**) Generation of the cover image using ODNMI.

A comparison between Figure 3a,b reveals that the number of rows in the original image and the cover generated using ODNMI are the same, thus reducing the redundancy by half compared to conventional interpolation-based methods. Seed pixels (pixels of the original image) are retained, with their value, in the cover image, without any modification, and remain unaltered throughout the whole embedding process. These pixels are later used to extract the original image, thereby fulfilling the utmost requirement of RDH.

### 3.2. AES-128 Encryption and Data Shifting

The security of secret data is a priority in cases of sensitive applications, including healthcare. Various data breaches are noted each year in the healthcare sector, demanding the development of novel mechanisms to enhance security and privacy. In the proposed scheme, we encrypted the secret data using an AES-128 encryption scheme to enhance its security. It is a symmetric cipher that involves ten rounds, pertaining to substitution and transposition of the secret information. In this work, we considered a key length of 128 bits, due to the advantage of faster encryption. After encrypting the data, Left Data Shifting (LDS) was used to embed the data into the cover image. It is a novel approach to hiding more data while observing less deterioration of the image. The data to be embedded is first converted into 3-bit (000 through 111) chunks, representing a decimal value ranging from zero through seven. The data, including the entire range of digits that were converted to 3-bit chunks, are then shifted four steps towards the negative axis of the number line; i.e., zero through seven yields negative four through three (100 through 011). Hence, the digits “0, 1, 2, 3, 4, 5, 6, and 7”, after a four-step shift towards negative axis of the number line, are substituted by “−4, −3, −2, −1, 0, 1, 2, and 3”, respectively. A higher number of bits are needed to represent the magnitude of digits zero through seven, in comparison to the magnitude of digits negative four through three. Thus, embedding data in the resultant format would yield a less deteriorated cover image when compared to conventional methods. The undesirable pixel values (out of range pixel values due to the addition of decimal values corresponding to 3-bit message chunks) are taken care of by setting different boundary conditions, so that the value of resultant pixels falls within the grayscale range of 0 to 255.

### 3.3. Data Embedding

This sub-section discusses the embedding of secret data, along with a fragile watermark, in the cover/host image generated using ODNMI. The secret data, after concatenating with the watermark, is embedded in the designated pixels while leaving seed pixels un-altered. The embedding was tested with two different methods, and is thus explained as two different techniques: Scheme-1 and Scheme-2. In the former technique (Scheme-1), data are embedded directly using LSB substitution, while as in the latter technique, before LSB embedding, the data are first subjected to left data shifting (LDS). For a comprehensive comparison, both techniques are discussed in detail. For authentication purposes, a 64 × 64 fragile watermark was interleaved with the data to be embedded in such a way that after every 47 bits of secret data, one watermark bit is embedded. The interleaving of the watermark bits throughout the image ensures high sensitivity to attacks, and, as such, any malicious attack to a block in the stego image is detected. The secret binary data, along with the interleaved watermark bits, after concatenating into a single row vector, are converted to a three-bit column vector. The 3-bit data chunks are then converted to corresponding decimal equivalents which yield values of zero through seven. These decimal equivalents are then embedded in the cover, using either of the following two techniques:

#### 3.3.1. Technique-1: Direct Embedding

In this technique, before embedding, we ensure that the three least significant bits of the pixel meant for embedding are set to zero, and that the decimal equivalent is then added to the pixels value. All 65,536 decimal equivalents are embedded in their respective pixels, thus embedding 196,608 data bits. This technique yields a maximum PSNR of 40.95 dB, with a payload of 196,608 bits (1.5 bpp). The average time required for embedding the data is 1.86 s.

#### 3.3.2. Technique-2: Embedding with LDS

In this technique, the data are first subjected to LDS, so that all decimal equivalents are shifted left by four steps; the data ranging from zero through seven, before LDS, range between negative four and three after LDS. As discussed earlier, in Section 3.2, LDS reduces the probability of changing the value of a pixel by a wider margin than embedding directly with LSB substitution, and thus leads to a better stego-image. With LDS, some boundary conditions do arise during the process, which are taken care of in such a way that, after any arithmetic operation, the pixel becomes confined within its designated value as explained by Equations (4)–(6).

(4)  C(i,j)=C(i,j) for 3<C(i,j)<253

(5) C(i,j)=C(i,j)+4 for C(i,j)≤3

(6) C(i,j)=C(i,j)−3 for C(i,j)≥253

The payload in this technique remains the same as in Scheme-1 (1.5 bpp), but a considerable improvement is observed in the imperceptibly, with PSNR raised from 40.95 dB to 43.75 dB, thus contributing to the perceptual quality enhancement of the stego image. In comparison to Scheme-1, this technique takes less time to embed the same amount of secret data; the average data embedding time is slightly reduced to 1.408 s. Algorithm 1 describes in detail the various steps involved in data/watermark embedding.
**Algorithm 1:** Watermark embedding in Cover-Image**INPUT: Original Image “I”, Watermark, Secret Data***Step: 1 Take an input image “I” of size M × N.**Step: 2 Generate cover “C” of size M × 2N, by interleaving columns at alternate levels using ODNMI.**Step: 3 Take a fragile watermark sized (n × n).**Step: 4 Convert the watermark to a single row vector.**Step: 5 Take the secret data as a bitstream of a single-row vector and interleave the watermark bits in the data at regular intervals. In the proposed case one watermark bit is being interleaved after every 47 secret data bits. Apply AES-128 encryption to the secret data.**Step: 6 Covert all three-bit chunks into corresponding decimal equivalents by using binary to decimal conversion.**Step: 7 For Technique 1: Set three LSBs of all data pixels to zero, excluding seed pixels.**For Technique 2: Apply LDS to the resultant decimal equivalents.**Step: 8 Add the decimal equivalents to the corresponding data pixels.**Step: 9 Repeat Step 8 till all decimal equivalents of the secret data are embedded to obtain stego-image.***OUTPUT: Stego Image “S”**

### 3.4. Data Extraction

The data extraction process is the reverse of data embedding. The extraction is comprised of two parts; one is the extraction of the original image, and the second the extraction of the secret data interleaved with a fragile watermark. For extraction of the seed image, the unaltered seed pixels of the cover image are used, while for data extraction a reverse process of data embedding is used. The technique involved in embedding will decide the extraction of secret data. Algorithm 2 presents a stepwise extraction process for both cases.
**Algorithm 2:** Watermark and secret data extraction from Stego-Image**INPUT: Stego Image “S”***Step: 1 Let “S” be the received stego image sized (M × 2N).**Step: 2 Extract the original image “I” of size (M × N) from the seed pixels.**Step: 3**Technique-1: Extract the data embedded in the three least significant bits of each pixel.**Technique-2: Reverse the boundary conditions deployed during the process of embedding and extract the last three bits from each non-seed pixel**Step: 4 Save the decimal equivalents in a data extraction row matrix.**Step: 5 Convert the matrix into a binary row with the help of decimal to binary conversion.**Step: 6 Reshape the matrix to a 48-column matrix and extract the 48th column, which contains the data of the fragile watermark.**Step:7 Reshape the 48th column bits obtained in step 6 into a (64 × 64) binary matrix, which is then extracted fragile watermark.**Step:8 Reshape the remaining 47 columns matric obtained in step 6 into a row matrix to yield the embedded secret. Apply AES-128 decryption to obtain the actual secret information.***OUTPUT: Original Image “I”, Watermark “W”, and Secret Data “D”**

## 4. Results and Discussion

**Figure 4:**
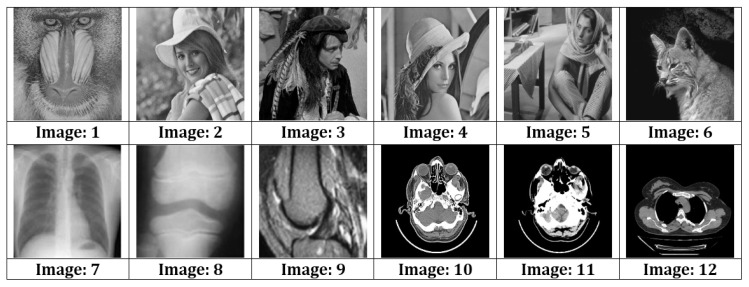
Various test images.

**Figure 5:**
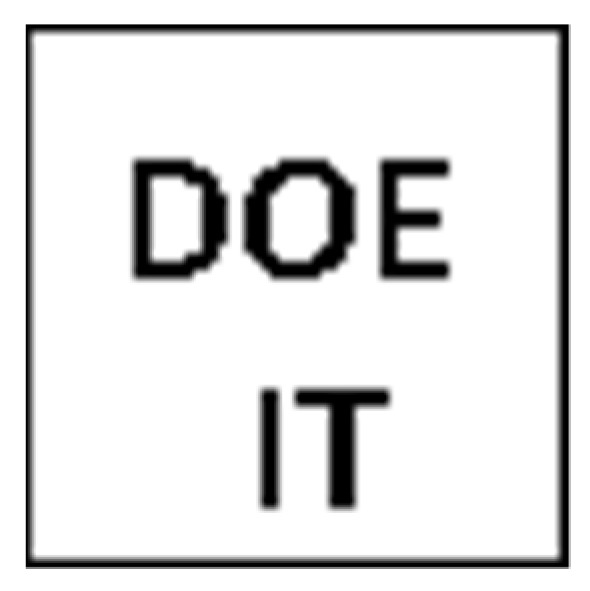
Watermark.

This section presents the results obtained during experimentation, and discussion of those results. Various commonly used test images, as shown in Figure 4 (Images 1–12), were used to test the proposed system. Six of the images (1 to 8) were obtained from open-source image databases (USC-SIPI, 2017, https://sipi.usc.edu/database/database.php?volume=misc, accessed on 23 January 2022); Open Access Biomedical Image Search Engine (OPENi, 2018; Image Processing Place, 2017, https://openi.nlm.nih.gov/, accessed on 23 January 2022), while four medical images (9 to 12) were obtained from a local diagnostic center (for education and research purposes). A 64 × 64 binary digital watermark, shown in Figure 5, was used for authentication purposes.

The experiment was carried out using MATLAB. We tested both the schemes using subjective as well as objective parameters. The objective quality metrics used to analyze the schemes include Peak Signal to Noise Ratio (PSNR) and Structural Similarity Measure Index (SSIM) [25]. For content authentication, parameters such as Bit Error Rate (BER %) and Normalized Cross-Correlation (NCC) were used.

### 4.1. Imperceptibility Analysis

Imperceptibility refers to the relative quality of the image (embedded with the secret data) compared to the original image, and is one of the most important parameters used to check the efficiency of a data-hiding scheme. The stego-image resulting after data embedding should be such that it should not draw attention to the adversary. The PSNR, calculated from the differences between the original, cover, and stego-images, provides an idea about the quality of the watermarked images. A high PSNR value indicates good visual quality, and effectiveness of the in embedding scheme. The objective and subjective quality results, for both techniques, under no attack, are shown in Table 1.

Table 1 presents various quality metrics for various test images, for both of the proposed schemes. The minimum PSNR of the former is 38.947 dB, with an average value of 40.33 dB. The use of LDS significantly improves the quality of stego-images, as can be seen from the results. On average, it improves the PSNR by a significant margin of about 3 dB. The average PSNR for the two schemes, at an embedding capacity of 1.5 bpp, was 40.338 dB and 43.75 dB, respectively. A comparison of results of the two proposed schemes and several state-of-the-art techniques is depicted below, in Table 2. Results reveal that the proposed scheme offers better quality stego-images, while maintaining reversibility and a higher embedding capacity. It is pertinent to mention that Lee et al.’s scheme offers more embedding capacity than the proposed scheme, but the PSNR is significantly lower.

### 4.2. Reversibility Analysis

**Figure 6:**
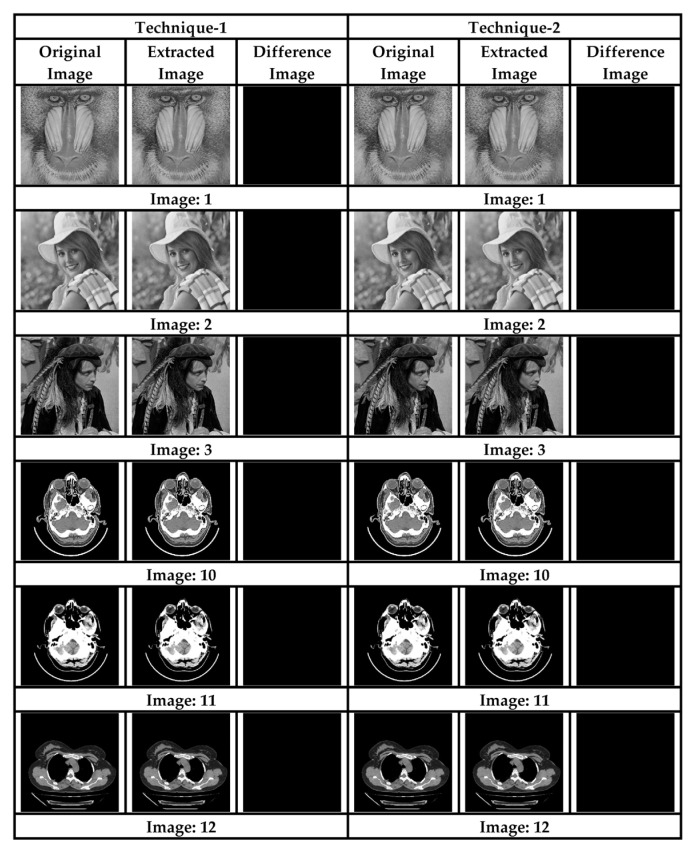
Subjective analysis for reversibility.

In the proposed schemes, the original image is recovered from the seed pixels after the extraction of data from the corresponding stego images. A difference between any pixel set of the two images (cover and stego), for both the schemes, results in a perfect black image, thus ensuring that the corresponding pixel difference between the two images is zero, as depicted in Figure 6. The subjective results, shown in Figure 6, show that both of the proposed schemes are perfectly reversible.

### 4.3. Computational Complexity and Memory Usage

As already discussed in Section 3.1, ODNMI is a one-dimensional version of NMI, and requires exactly one half of the computational operations required by NMI. This is because it uses only one-dimensional interpolation, either in the horizontal direction or the vertical direction. Thus, it is quite intuitive that the proposed scheme has reduced computational complexity compared to various two-dimensional state-of-the-art interpolation schemes, such as NMI, INP, PRM, OPR, and PTB. Further, we present the time taken by the proposed algorithm for data embedding in Table 3. It is evident from the table that the proposed scheme takes an average time of 0.4459 s for the embedding phase.

Memory usage is another parameter of huge importance when discussing the transmission of data over channels with limited bandwidth. Memory usage of the proposed algorithm is reduced by 50%, as can be seen in Table 4, which presents data pertaining to the memory requirements of a conventional NMI technique and the novel ODNMI technique. The transmission of a 512 × 512 image requires a memory of 1024 KB, using conventional NMI, while it requires only 512 KB using the proposed ODNMI technique, making the proposed technique a superior candidate in terms of both memory usage and time consumption.

### 4.4. Authentication Analysis

**Figure 7:**
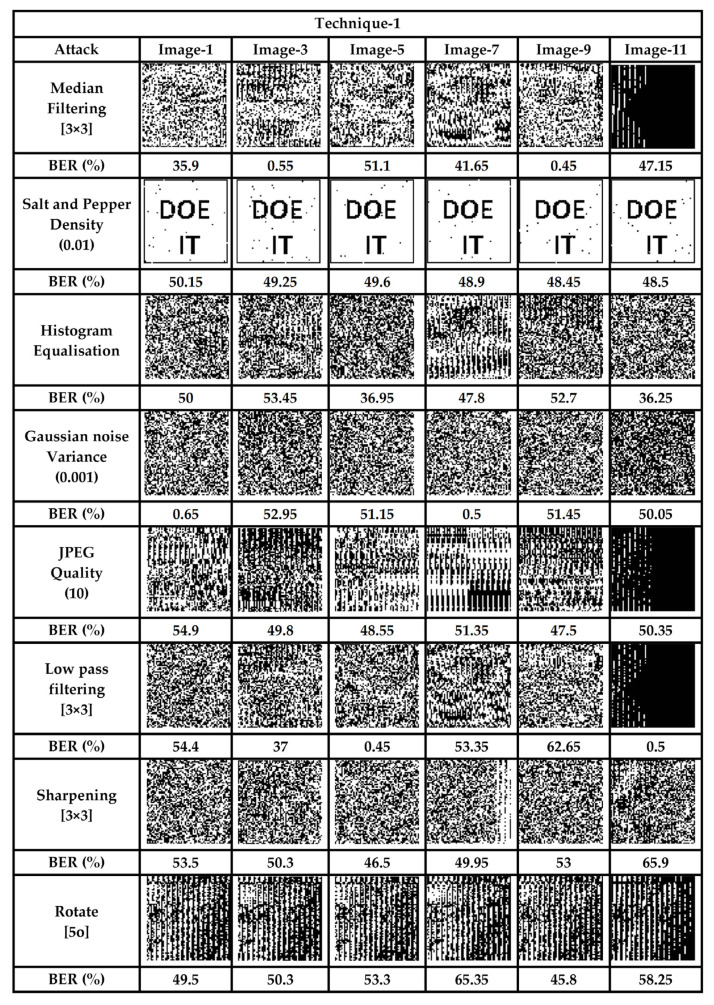
Authentication and fragility analysis (Technique-1).

**Figure 8:**
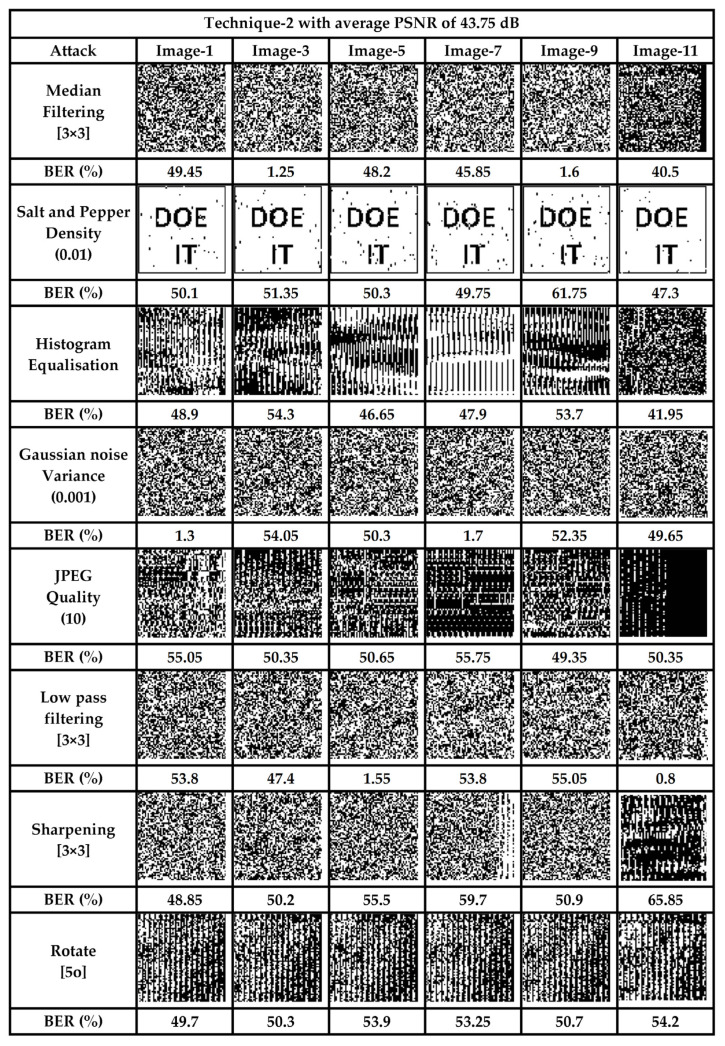
Authentication and fragility analysis (Technique-2).

Visual-IoT communication channels are usually insecure, and when image data are transmitted from one node to another there are chances for it being tampered with, as a result of various intentional and unintentional attacks. For a reversible data-hiding technique, extraction of the original image and secret data are the prime requirements. An erroneous extraction as a result of an intentional /un-intentional attack leads to unfaithful retrieval. For authentication of both the images and the secret data, a fragile watermark is embedded into the cover image. This watermark is first extracted from the stego-image at the receiving end, and then compared to the embedded one, to facilitate authentication. If the two watermarks, embedded and extracted, are the same, the reception is considered error-free. Since, in this technique, we have interleaved watermark bits throughout the whole cover image (one bit after every 47 data bits), tampering with any part of the image is reflected at the output. Various signal processing and geometric attacks were used to test the proposed scheme. It is pertinent to mention here that all the stego images (Image-1 through Image-12) were tested with both techniques, using the aforementioned attacks. However, due to space limitations, we have arbitrarily selected some of the images, as presented in Figure 7 and Figure 8.

From Figure 7 and Figure 8, it is evident that any tampering with the stego-image results in extraction of the unrecognized watermark, hence validating the fact that the scheme is sensitive to various signal processing and geometrical attacks carried out on it. The average BER in the proposed scheme, to various attacks carried out on stego images, was 43.91% and 45.14% for the two techniques, respectively, which ensures that any attack on the data during transit would be successfully detected.

In addition to the subjective analysis, we also carried out an objective analysis of the two schemes, presented for various attacks, such as salt and pepper noise addition and median filtering, for the whole set of images. The objective quality metrics obtained by subjecting the stego-images to different attacks are presented in Table 5. For an attacked image, the SSIM yields a very low value, while the BER results in a higher value. The higher BER values and lower SSIM values, along with the PSNR, show that the scheme is highly fragile, and hence capable of detecting any tamper. Table 5 and Table 6 present the exact count of errors for a payload of 1.5 bpp, along with various objective quality metrics for technique-1 and -2, respectively.

### 4.5. A Brief Discussion of the Results

In this sub-section, we present a brief discussion regarding the results obtained for SVIoT. We evaluated SVIoT for imperceptibly, reversibility, content authentication, and computational complexity. It was shown that the proposed scheme is capable of providing high-quality stego-images. The rate of distortion was significantly improved (by 3 dB) when LDS was used. The average PSNR was greater than 43 dB for a payload of 1.5 bpp, while maintaining reversibility, which is a significant result, and is superior to the results of [21,22,23,25,27]. Although [12] provided a superior PSNR, unlike the proposed scheme, their technique was not able to detect tamper, a significant parameter of importance in critical applications, such as healthcare, driven by visual-IoT. We fully demonstrated the reversibility of the proposed scheme in Section 4.2. In addition to authenticating the data received, we tested our system for various signal processing and geometric attacks, such as Gaussian noise, salt and pepper noise, median filtering, low pass filtering, sharpening, JPEG compression, etc. The image quality parameters PSNR, SSIM, and BER were for evaluation. It was shown in detail in Section 4.3 that all attacks carried out on the stego-images reported a BER of more than 50%, signifying that the hidden fragile watermark was not faithfully received. This informs the receiver that the data has been attacked during transit. An IoT-based system is resource-constrained, and thus has lesser memory/bandwidth available. This demands that the processes carried out by these systems be less computationally complex. Towards this end, the significant contribution of this paper is the development and use of ODNMI. The use of ODNMI increases the computational efficiency of the system, as it requires only 50% of the operation and storage resources of well-known schemes, such as [21,22,23,25,27], while maintaining reversibility and a higher payload. Given all the attributes of the system, it is a potential candidate for security and authentication in visual-IoT-driven smart-health systems for futuristic sustainable computing.

## 5. Conclusions

The security of critical information (such as patient records, etc.) is of significant importance in a smart-city scenario. IoT-driven systems are resource constrained, and, as such, resource reduction is a premier concern when developing security algorithms for such systems. In this paper, we present a secure framework SVIoT for the novel multimedia concept of ODNMI. This approach reduces the computational and storage requirements by a significant factor of 50 percent. An original image of size M × N is up-scaled to M × 2N, which is dissimilar to conventional interpolation methods, wherein images are up-scaled to 2M × 2N. Secret data are encrypted using an AES-128 algorithm, to enhance security. Further, a novel mechanism, LDS, was used before embedding data in the cover image. LDS ensures better perceptual quality at a relatively high payload, as it encodes a three-bit data chunk into two-bit chunk, prior to embedding. We achieved an average PSNR of 43.75 dB for a payload of 1.5 bpp (bits per pixel) using the proposed scheme, which is superior to state-of-the-art techniques. The proposed RDH scheme allows the complete recovery of the original image, without any degradation, as well as the reduction of redundant data by 50 percent. Moreover, we embedded a fragile watermark in the cover image to ensure authentication of the received content. In the future, we aim to develop and test this scheme on an embedded system platform.

## Figures and Tables

**Table 1:** Objective quality indices for Technique-1 and Technique-2.

Image No	Technique-1 (without the Use of LDS)			Technique-2 (with the Use of LDS)		
	PSNR (dB)	SSIM	%BER	PSNR(dB)	SSIM	%BER
1.	40.089	0.979	0	43.73	0.989	0
2.	40.93	0.962	0	43.76	0.980	0
3.	40.982	0.969	0	43.74	0.984	0
4.	40.954	0.96	0	43.74	0.978	0
5.	40.958	0.97	0	43.75	0.983	0
6.	40.021	0.956	0	43.75	0.979	0
7.	40.923	0.942	0	43.75	0.968	0
8.	40.917	0.939	0	43.76	0.965	0
9.	40.953	0.966	0	43.75	0.981	0
10.	39.19	0.772	0	43.74	0.970	0
11.	38.947	0.751	0	43.76	0.967	0
12.	39.191	0.766	0	43.78	0.968	0

**Table 2:** Comparison to state-of-the-art techniques.

Scheme	Average PSNR (dB)	Capacity (bpp)
Jung et al. [25]	33.24	0.96
Lee et al. [24]	33.79	1.59
Parah et al. [30]	46.36	0.75
Naheed et al. [23] (GA Scheme)	49.01	0.15
Naheed et al. [23] (PSO Scheme)	49.00	0.15
Luo et al. [29]	48.94	0.14
Wahed and Nyeem [11]	47.61	1.5
Kaw et al. [27]	43.6	1.25
Proposed		
Technique-1	40.338	1.5
Technique-2	43.75	1.5

**Table 3:** Embedding time of the proposed scheme (in seconds).

Image	Embedding time (s)
Image: 1	0.4804
Image: 2	0.4316
Image: 3	0.4336
Image: 7	0.4472
Image: 8	0.4550
Image: 9	0.4277
Average	0.4459

**Table 4:** Memory usage of the NMI and proposed ODNMI schemes.

Original Image Size	Memory Needed to Store Stego-Image Using NMI	Memory Needed to Store Stego-Image Using ODNMI
512 × 512 = 256 KB	1024 KB	512 KB
256 × 256 = 64 KB	256 KB	128 KB
128 × 128 = 16 KB	64 KB	32 KB

**Table 5:** Authentication analysis for salt and pepper and median filtering (Techniques-1).

Attacked Stego Image	Tech-1 Average BER (%) = 43.916					
	Salt and Pepper Noise Density = 0.01			Median Filtering [3 × 3]		
	PSNR	SSIM Cover Attacked Stego	Erroneous Primary Data Bits	PSNR	SSIM Cover Attacked Stego	Erroneous Primary Data Bits
Image-1	25.59	0.798	949	28.8	0.803	76410
Image-2	25.32	0.733	956	39.1	0.963	96191
Image-3	24.44	0.783	1009	32.54	0.925	74547
Image-4	25.31	0.732	988	36.48	0.96	77441
Image-5	25.31	0.778	970	33.07	0.91	74960
Image-6	24.44	0.763	949	33.93	0.918	80290
Image-7	25.27	0.668	911	44.21	0.985	84888
Image-8	24.99	0.668	933	42.67	0.979	87739
Image-9	25.11	0.743	1031	40.63	0.984	72935
Image-10	23.65	0.647	960	26.7	0.846	85542
Image-11	23.23	0.613	1015	31.06	0.885	87244
Image-12	23.08	0.611	914	29.11	0.861	86083
Av. Values	24.64	0.711		34.85	0.918	

**Table 6:** Authentication analysis for salt and pepper and median filtering (Techniques-2).

Attacked Stego Image	Tech-2 Average BER (%) = 45.148					
	Salt and Pepper Noise Density = 0.01			Median Filtering [3 × 3]		
	PSNR	SSIM Cover Attacked Stego	Erroneous Primary Data Bits	PSNR	SSIM Cover Attacked Stego	Erroneous Primary Data Bits
Image-1	25.9	0.815	2840	28.8	0.806	93,582
Image-2	25.38	0.747	2795	39.49	0.967	88,322
Image-3	24.44	0.792	2981	32.54	0.921	90,932
Image-4	25.49	0.753	2771	36.72	0.964	90,184
Image-5	25.1	0.777	2871	33.16	0.913	88,876
Image-6	24.22	0.772	2346	34.2	0.93	94,053
Image-7	25.27	0.682	2815	45.02	0.99	88,238
Image-8	24.93	0.681	2903	43.39	0.986	89,726
Image-9	25.22	0.753	2929	41.03	0.987	77,294
Image-10	23.41	0.792	2017	26.57	0.793	96,549
Image-11	23.39	0.784	1866	30.62	0.823	95,625
Image-12	23.69	0.781	2026	28.85	0.804	94,576
Av. Values	24.70	0.760		35.03	0.907	

## Data Availability

Open-source image databases (USC-SIPI, 2017, https://sipi.usc.edu/database/database.php?volume=misc, accessed on 23 January 2022); Open Access Biomedical Image Search Engine (OPENi, 2018; Image Processing Place, 2017, https://openi.nlm.nih.gov/, accessed on 23 January 2022).
